# Improved 3D U-Net robustness against JPEG 2000 compression for male pelvic organ segmentation in radiotherapy

**DOI:** 10.1117/1.JMI.8.4.041207

**Published:** 2021-04-05

**Authors:** Karim El Khoury, Martin Fockedey, Eliott Brion, Benoît Macq

**Affiliations:** Université Catholique de Louvain, Institute of Information and Communication Technologies, Electronics and Applied Mathematics, Louvain-La-Neuve, Belgium

**Keywords:** U-Net segmentation, JPEG2000, convolutional neural networks, image compression, 3D medical imaging

## Abstract

**Purpose:** Automation of organ segmentation, via convolutional neural networks (CNNs), is key to facilitate the work of medical practitioners by ensuring that the adequate radiation dose is delivered to the target area while avoiding harmful exposure of healthy organs. The issue with CNNs is that they require large amounts of data transfer and storage which makes the use of image compression a necessity. Compression will affect image quality which in turn affects the segmentation process. We address the dilemma involved with handling large amounts of data while preserving segmentation accuracy.

**Approach:** We analyze and improve 2D and 3D U-Net robustness against JPEG 2000 compression for male pelvic organ segmentation. We conduct three experiments on 56 cone beam computed tomography (CT) and 74 CT scans targeting bladder and rectum segmentation. The two objectives of the experiments are to compare the compression robustness of 2D versus 3D U-Net and to improve the 3D U-Net compression tolerance via fine-tuning.

**Results:** We show that a 3D U-Net is 50% more robust to compression than a 2D U-Net. Moreover, by fine-tuning the 3D U-Net, we can double its compression tolerance compared to a 2D U-Net. Furthermore, we determine that fine-tuning the network to a compression ratio of 64:1 will ensure its flexibility to be used at compression ratios equal or lower.

**Conclusions:** We reduce the potential risk involved with using image compression on automated organ segmentation. We demonstrate that a 3D U-Net can be fine-tuned to handle high compression ratios while preserving segmentation accuracy.

## 1 Introduction

Radiotherapy cancer treatment is essentially made up of two phases: treatment planning and delivery. During the planning phase, which is done once at the beginning of the treatment, a computed tomography (CT) scan is taken, and after visual inspection, physicians manually outline the target and the surrounding healthy organs to compute a specific dose distribution. During the delivery phase, which is done daily for a period of up to 20 days, a cone beam computed tomography (CBCT) scan is acquired to determine the specific position in which a patient should be aligned before delivering each fraction of the required dose. Dose fractionation limits the patient’s health risks due to sudden large exposures. The process allows healthy cells to recuperate in time for the next dose delivery. The daily variations of organ size, shape, and position in the pelvic region are considerably large due to perpetual tasks such as filling and voiding the bladder and rectum. Given that both CT and CBCT scans are generated by exposing the patient to a substantial dose of radiation, detecting these variations becomes essential to avoid exposing healthy vital organs to large doses. The automation of the segmentation process is key to facilitate the work of medical practitioners, as manual organ segmentation is very time consuming (ranging from 2 to 4 h). Recent papers have addressed the segmentation of male pelvic organs using CT and CBCT scans via a deep learning approach. Schreier et al.^1^ use artificially-generated CBCT scans in their training set to segment male bladder, rectum, prostate, and seminal vesicles. Léger et al.^2^ implement a U-Net segmentation scheme that is trained on real CBCT scans to segment organs in the male pelvic region with high efficiency.

However, the issue with deep learning networks is that they require large amounts of training data. To reach the amount of required data, multi-site data collection is often mandatory. Therefore, image compression becomes a necessity when handling such huge amounts of data transfer and storage. Lossy compression such as JPEG 2000 is very attractive as it can reach high compression ratios. Nonetheless, lossy image compression will affect image quality which in turn can affect the efficiency of deep learning tasks and can therefore impact medical practitioners decision making. It is worth noting that compressing medical images is permitted by governing bodies in North America, the European Union, and Australia provided they do not affect the diagnostic capabilities of medical practitioners.^3^

Quantifying the diagnostic capabilities of physicians on compressed image is complex given that evaluations can be subjective and can vary depending on the task at hand. Several studies have yielded different compression ratio thresholds for different use cases in medical imaging. Krupinski et al.^4^ showed that when going up to compression ratios higher than 32:1, pathologists capability to differentiate breast cancer development stages was affected. Pantanowitz et al.^5^ demonstrated that in the case of the measurement of the HER2 score in images of breast carcinoma, a 200:1 compression ratio was accepted. In the work of Marcelo et al.,^6^ 10 different datasets of pathological images (mainly carcinomas and adenocarcinomas) are used to evaluate the impact of data compression on telepathology. A 95% accuracy was shown following testing with 10 medical practitioners of uncompressed and 90:1 JPEG compressed images. In the work of Kalinski et al.,^3^ a compression ratio of 20:1 was considered adequate to detect Helicobacter pylori gastritis from histopathological images. However, these studies assessed the impact of compression through physicians subjective evaluation criteria. Even though this evaluation criteria is definitely valid, it can become expensive and impractical when handling large datasets and complex deep learning tasks as medical practitioners time resources are scares. The introduction of high complexity models such as convolutional neural networks (CNN) into modern day medical imaging would impose a change in the evaluation criteria depending on the deep learning task at hand, whether it is classification or segmentation of medical images. Given that the CNN would be trained on compressed data, it yields a high tolerance to the distortion caused by image compression. A recent study by Zanjani et al.^7^ has validated the above point. They studied the impact of JPEG 2000 on CNN for classification of metastases in breast lymph node using histopathological whole side images. They showed that training their network on uncompressed images and testing it compressed images showed good performances up to a compression ration of 24:1. Meanwhile, training their network on compressed images at a ratio of 48:1 maintained correct detection performance for compression ratios of 48:1 and lower.

Studying the impact of image compression on deep learning-based U-Net segmentation is very valuable to both the computer science and medical imaging communities. Sharma et al.^8^ have analyzed the performance of semantic segmentation algorithms when trained with JPEG compressed images. Their results show that a network trained with a compressed dataset outperforms networks trained on uncompressed datasets. They also show that JPEG compression behaves as a data augmentation medium to improve semantic U-Net segmentation. To the extent of our knowledge, the impact of image compression on deep learning-based U-Net segmentation in medical imaging has yet to be addressed. In this paper, we propose to study the impact of JPEG 2000 on 2D and 3D CNN-based segmentation. More precisely, we worked on the robustness of 2D and 3D U-Net segmentation of male pelvic organs to JPEG 2000 compression. The U-Net architecture is a type of CNN typically used in medical imaging that has been modified to work with smaller datasets and yield higher segmentation accuracy. This research offers three main contributions on the impact of image compression on deep learning-based segmentation. (1) We show that a 3D U-Net segmentation in more resilient to high compression ratios than a 2D U-Net segmentation when trained on uncompressed data. (2) By fine-tuning the 3D U-Net segmentation, we can further increase its resilient to high compression ratios while maintaining the same segmentation performances with respect to the 2D U-Net. (3) We determine that fine-tuning our 3D network to a specific compression ratio will ensure its flexibility to be tested at compression ratios equal or lower with equivalently high performances.

This paper is organized as follows. In Sec. 2, we present our dataset and the methods used by presenting the JPEG 2000 compression algorithm, the 2D and 3D U-Net segmentation architectures, the evaluation metrics as well as the three experiments that have been conducted. In Sec. 3, we present the detailed individual results of the three experiments: 2D versus 3D U-Net robustness to compression, 3D U-Net fine-tuning and 3D U-Net training phase fine-tuning. In Sec. 4, we discuss on the previously obtained results for 2D and 3D U-Net segmentation, we position our results with respect to the literature and comment on key limitations that we faced in this study. In Sec. 5, we conclude by summarizing the outcomes of our paper and looking ahead at potential future work.

## 2 Materials and Methods

### 2.1 Dataset

The patients’ CTs and CBCTs come from the the CHU-UCL-Namur and CHU-Charleroi Hôpital André Vésale Belgium. André Vésale provided 56 CBCT, and 22 CT volumes while the CHU-UCL-Namur provided 52 CT volumes; all are from male patients. All the images were first stocked under the DICOM format after their acquisition. To create volumes from the 2D CT images, the CT slices were concatenated. Afterward all volumes were re-sampled to obtain a 1.2×1.2×1.5  mm regular grid in 192×192×160 matrices. This step was necessary to have equivalent size and scale for both CT and CBCT volumes. These matrices use a grayscale with integer values between 0 and 255. For each patient, three masks representing the bladder, the rectum and the background were annotated manually by a trained specialist. The training set is composed of 60 CTs and 45 CBCTs and the test set is composed of 14 CTs and 11 CBCTs, this partition gives a ratio of 80:20 for both data types. Before feeding the data into the networks, each volume was normalized using the mean and standard deviation of the training set.

### 2.2 JPEG 2000 Algorithm

After the creation of JPEG standard, JPEG 2000 was proposed as a follow-up standard having better rate-distortion performances and having additional features such as quality scalability.^9^ The main difference between the two algorithms is that JPEG 2000 uses the discrete wavelet transform (DWT) instead of the classical discrete cosine transform (DCT) used in JPEG. Even though the performances of JPEG and JPEG 2000 are quite similar at low compression ratios, JPEG 2000 outperforms JPEG at high compression ratios making it a key factor in the creation of the DICOM standard.^10^ In this paper, to be able to fix a target compression ratio on our dataset, the Python glymur library (which contains an interface to the OpenJPEG library) was used. Even though our dataset is composed of volumes, we compressed slices of this volumes as if they were images.

### 2.3 U-Net Segmentation Architecture

**Fig. 1:**
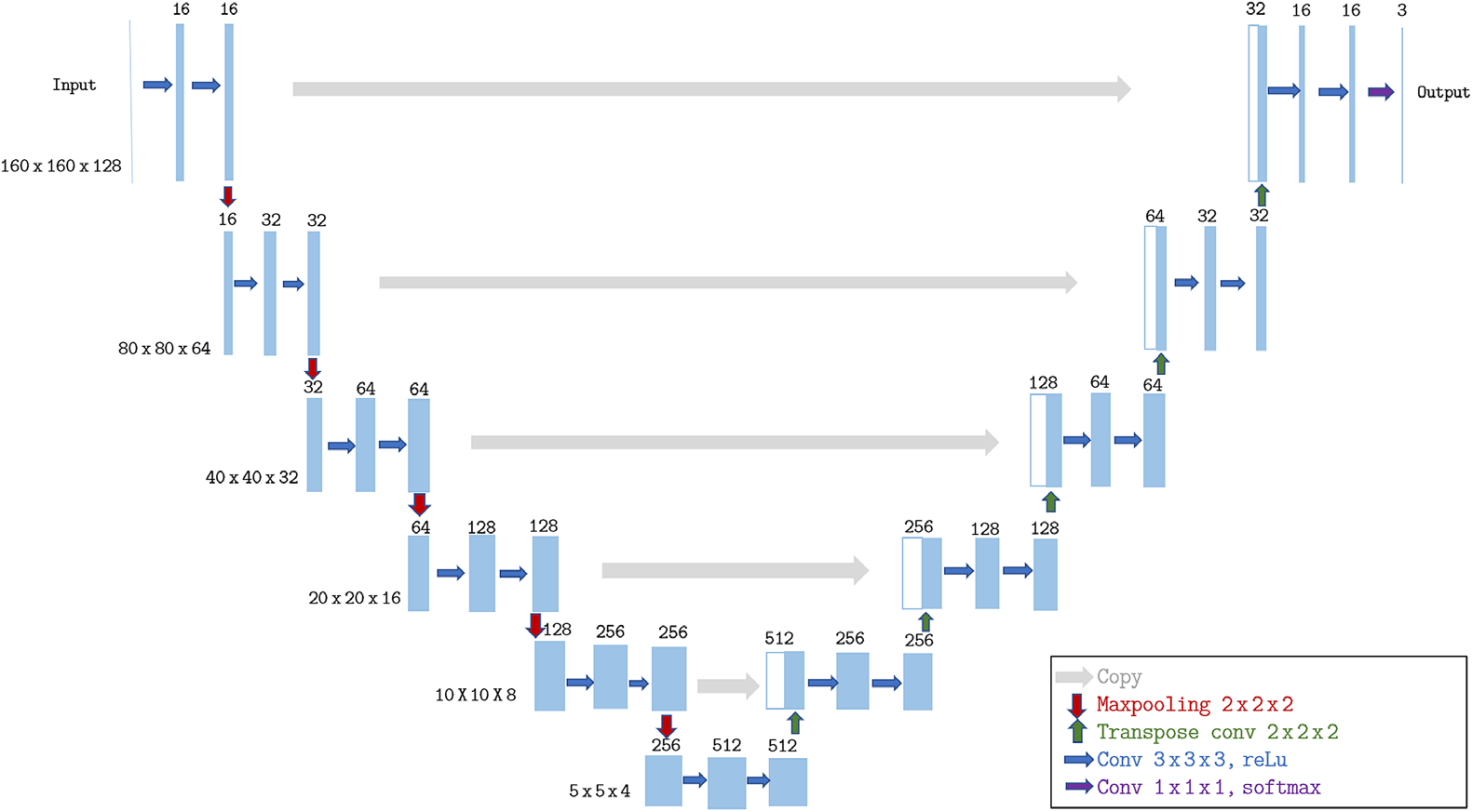
3D U-Net architecture scheme.

Two different architectures are considered in this paper: 3D U-Net and the 2D U-Net. The code implementing these architecture was adapted from the Github repository in Ref. 11 for the 2D U-Net and from the Github repository in Ref. 12 for the 3D U-Net. Both networks are similar in their structure, except for the following differences: (i) the 3D uses an additional dimension at all levels, (ii) at each level the 2D has four times more feature-maps than the 3D, (iii) the 3D network uses an additional pooling/up-convolution, and (iv) the volumes were re-scaled for the 3D from 192×192×160 to 160×160×128 to fit GPU memory constraints. The 3D U-Net network is shown in Fig. 1.

As first described by Ronneberger et al.,^13^ the U-Net is considered as a reference configuration for segmentation. This network uses a contracting path to extract contextual information and create deep features while forwarding higher resolution features in a up-scaling path to obtain an output with more detailed and complex edges. The 3D network (respectively 2D network) uses 3×3×3 convolution (respectively 3×3) with zero padding to preserve the dimensions of the feature maps. In the down-sampling path max pooling with stride 2 is used between each resolution level and the number of feature-maps is doubled. While in the up-sampling path, we halve the number of feature maps at each scale using 2×2 (respectively 2×2×2) up-convolutions.

### 2.4 Metrics and Loss Functions

We use $T_{i}$ and $t_{i,(x,y,z)}$ (respectively $P_{i}$ and $p_{i,(x,y,z)}$) as the targeted (respectively predicted) mask i and the targeted (respectively predicted) voxel values, these variables are used to define the different evaluation metrics and loss functions. The target is the area segmented by the medical specialist and the prediction is the network output. The index i refers to the segmentation class (0 = bladder, 1 = rectum, and 2 = background).

The Dice similarity coefficient (DSC) measures the quality of the segmentation by comparing the target and prediction common area to their volume size.

$$DSC_{i}=\frac{2\cdot |P_{i}\cap T_{i}|}{\left|P_{i}|+|T_{i}\right|}.$$ (1)

$$DSC_{i}=\frac{2\cdot \underset{x,y,z}{\sum /}p_{i,(x,y,z)}\cdot t_{i,(x,y,z)}}{\underset{x,y,z}{\sum }p_{i,(x,y,z)}+t_{i,(x,y,z)}}.$$ (2)

The symmetric mean boundary distance (SMBD) is a measure of the distance between the mask border. If we consider the distance between a point $p_{i,b}$ belonging to the predicted border $P_{i,b}$ and the target border $T_{i,b}$, we can express it as

$$d(p_{i,b},T_{i,b})=\underset{t\in T_{b}}{\min}{\left\|s⊙(p_{i,b}-t_{i,b})\right\|}_{2}.$$ (3)

${\left\|.\right\|}_{2}$ denotes the euclidean norm and $s^{t}$ = (1.2, 1.2, 1.5) is the pixel spacing in mm. Then, we can measure the mean boundary distance.

$$D(P_{i,b},T_{i,b})=\frac{1}{n}\underset{p_{i,b}}{\sum }d(p_{i,b},T_{i,b}).$$ (4)

This metric is asymmetric as $D(P_{i,b},T_{i,b})\neq D(T_{i,b},P_{i,b})$, it is why we use the SMBD,

$$SMBD=\frac{D(P_{i,b},T_{i,b})+D(T_{i,b},P_{i,b})}{2}.$$ (5)

The loss function $L_{3D}$ used for the 3D U-Net is defined in Eq. (6), it is based on the opposite of the DSC of the organs. The uneven volumes of the classes created convergence issues which were solved by weighting the three for the rectum DSC and discarding the background DSC.

$$L_{3D}=-DSC_{0}-3\cdot DSC_{1}.$$ (6)

The DSC could not be used on the 2D U-Net training because for some slices only one or even no organ was present creating problems for the batch DSC computation. Thus, we opted for the cross-entropy function $L_{2D}$ as loss function,

$$L_{2D}=-\underset{i=0,1,2}{\sum }\underset{x,y}{\sum }t_{i,(x,y)}\;\log(p_{i,(x,y)}).$$ (7)

To compare the 2D and 3D network performances rigorously, the DSC results shown will always be computed based on 3D matrices. Thus for the 2D network each slice will be predicted individually and then concatenated with the others of the same volume before calculating its DSC.

### 2.5 Experiments

**Fig. 2:**
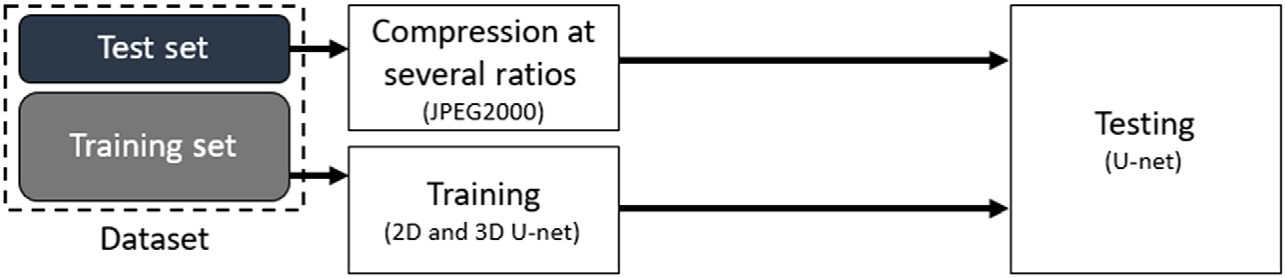
Experiment 1 block diagram: 2D versus 3D U-Net robustness to JPEG 2000 compression at several compression ratios.

In this work, we set up three experiments to determine the optimal way to train the CNN to be robust against the impact of JPEG 2000 compression:

In the first experiment, we compare the performances of 2D versus 3D U-Net segmentation on compressed images. Referring to the block diagram in Fig. 2, we train both the 2D and 3D U-Net networks on uncompressed images from the data set. We then compress/decompress the test set using JPEG 2000 at several compression ratios to evaluate the networks performances at each compression ratio.

**Fig. 3:**
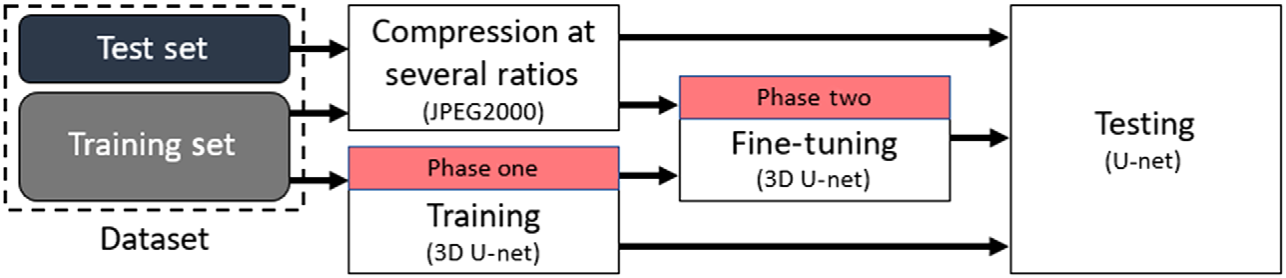
Experiment 2 block diagram: 3D versus fine-tuned 3D U-Net robustness to JPEG 2000 compression for training at several compression ratios and testing at the same compression ratios.

In the second experiment, we fine-tune the 3D U-Net training. Referring to the block diagram in Fig. 3, in addition, the first training phase where we train the network on uncompressed images, we add a second training phase where we train the network on compressed/decompressed images at several compression ratio and test them on the same compression ratio that the network was trained on.

**Fig. 4:**
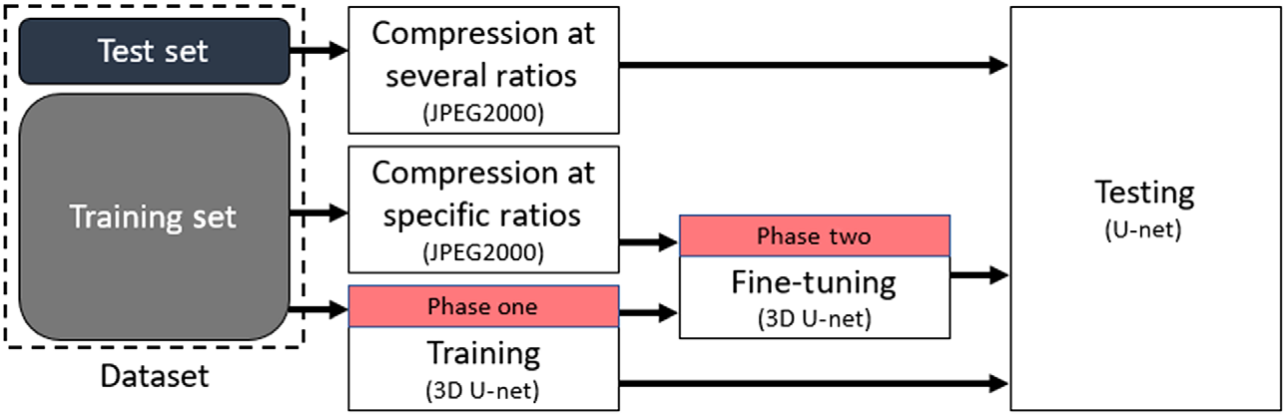
Experiment 3 block diagram: 3D fine-tuned at a specific compression ratio for robustness to JPEG 2000 compression and testing at several compression ratios.

In the third experiment, we look to improve the fine-tuning of the 3D U-Net training phase. Referring to the block diagram in Fig. 4, we use a similar approach to that of the second experiment, however, we train our network on one specific compression ratio and test it on several compression ratios. By training our network in this manner, we are able to determine the ideal compression ratio at which to store our images for training and testing of the 3D U-Net segmentation.

## 3 Results

### 3.1 Experiment 1: 2D Versus 3D U-Net Robustness to JPEG 2000 Compression Tested at Several Compression Ratio

In this experiment, we train both the 2D and 3D U-Net on uncompressed images and test them on compressed/decompressed images for each compression ratio ranging from 24:1 to 128:1. We then look at two segmentation evaluation metrics, the DSC and SMBD, in the case of both the CT and CBCT datasets on bladder and rectum segmentation. The detailed results of the experiment for both bladder and rectum segmentation are summarized in Tables 1 and 2, respectively.

**Table 1:** Experiment 1—Detailed DSC and SMBD performances at several compression ratios for 2D and 3D U-Net segmentation of the bladder on CT and CBCT images.

Bladder		Compression ratio						
		1:1	24:1	32:1	48:1	64:1	96:1	128:1
DSC	2D CT	0.918±0.039	0.914±0.038	0.902±0.042	0.872±0.067	0.809±0.088	0.578±0.182	0.301±0.260
	3D CT	0.916±0.033	0.912±0.034	0.902±0.043	0.885±0.060	0.859±0.076	0.758±0.122	0.409±0.291
	2D CBCT	0.855±0.068	0.842±0.071	0.805±0.074	0.748±0.073	0.651±0.078	0.347±0.077	0.175±0.096
	3D CBCT	0.832±0.118	0.825±0.123	0.808±0.129	0.780±0.138	0.741±0.141	0.651±0.118	0.450±0.133
SMBD	2D CT	2.040±1.404	2.251±1.280	2.407±1.170	3.157±1.455	4.663±2.546	7.204±3.598	10.08±2.741
	3D CT	1.445±0.579	1.546±0.615	1.831±0.806	2.083±1.123	2.458±1.356	3.901±1.995	8.205±4.186
	2D CBCT	2.883±1.827	3.015±1.702	3.490±1.699	4.121±1.662	4.912±1.336	7.989±2.466	13.20±4.251
	3D CBCT	2.582±1.963	2.695±2.033	2.955±2.167	3.361±2.364	3.857±2.371	4.902±1.920	7.811±2.151

**Table 2:** Experiment 1—Detailed DSC and SMBD performances at several compression ratios for 2D and 3D U-Net segmentation of the rectum on CT and CBCT images.

Rectum		Compression ratio						
		1:1	24:1	32:1	48:1	64:1	96:1	128:1
DSC	2D CT	0.751±0.093	0.755±0.090	0.728±0.091	0.668±0.114	0.576±0.159	0.370±0.155	0.135±0.091
	3D CT	0.774±0.068	0.772±0.066	0.773±0.061	0.752±0.062	0.709±0.063	0.617±0.094	0.373±0.186
	2D CBCT	0.780±0.071	0.756±0.083	0.708±0.106	0.641±0.114	0.524±0.132	0.283±0.147	0.071±0.062
	3D CBCT	0.766±0.073	0.765±0.070	0.748±0.078	0.711±0.097	0.647±0.120	0.471±0.114	0.272±0.135
SMBD	2D CT	3.760±1.029	3.537±1.027	3.402±0.977	3.646±1.294	3.875±1.555	5.296±2.128	9.811±4.374
	3D CT	2.653±1.034	2.642±1.056	2.602±0.967	2.862±1.146	3.197±1.065	4.322±2.432	8.917±4.143
	2D CBCT	2.730±1.085	2.837±1.088	3.057±1.160	3.223±1.073	3.690±1.022	4.977±1.160	8.802±2.425
	3D CBCT	2.348±0.867	2.308±0.802	2.413±0.823	2.604±0.952	3.245±1.593	4.935±1.689	9.404±2.837

**Fig. 5:**
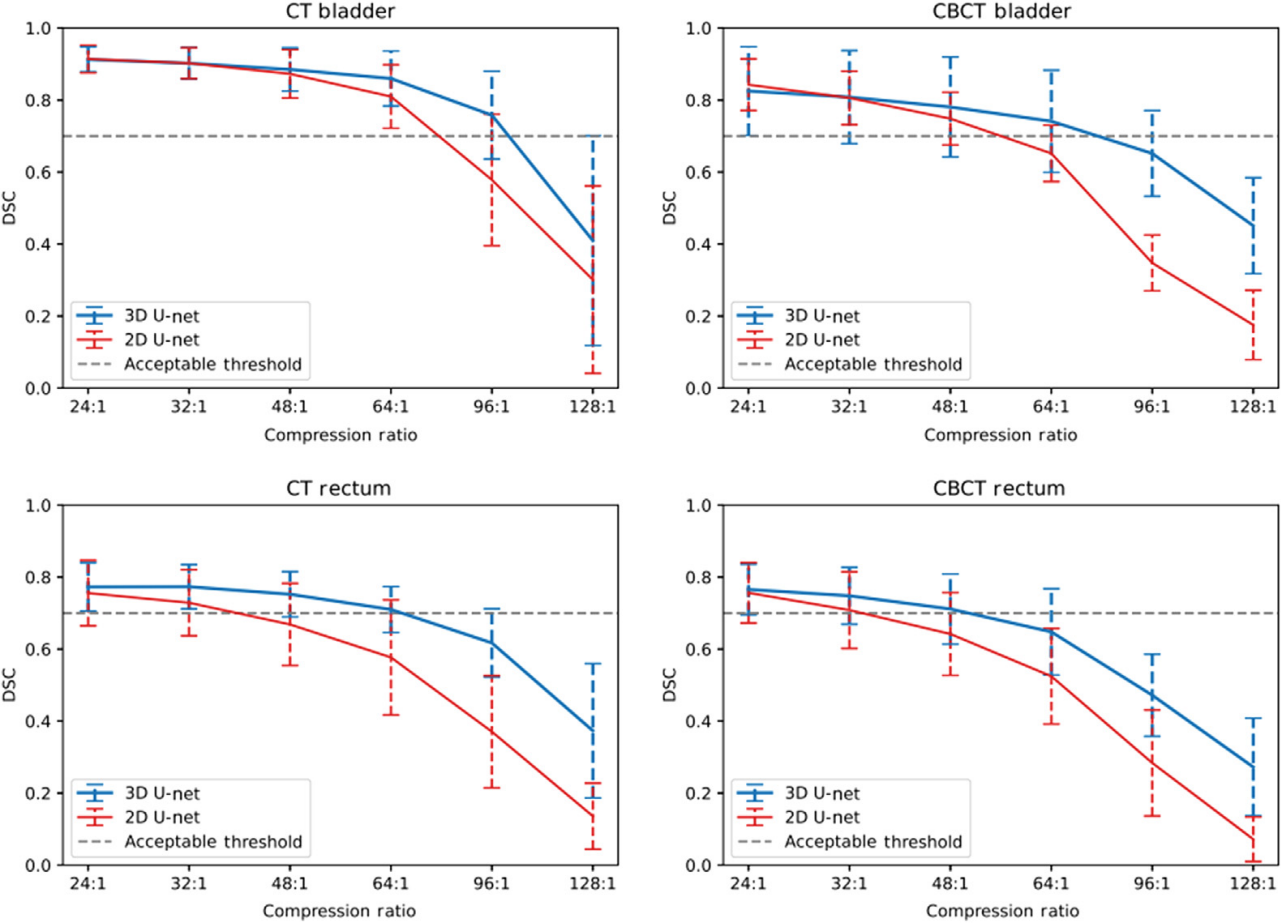
Experiment 1—DSC performances at several compression ratios for 2D (red) and 3D (blue) U-Net segmentation of the bladder (top) and the rectum (bottom) on CT (left) and CBCT (right) images.

Referring to Fig. 5, we can observe better overall performance of the 3D U-Net network in terms of the DSC when testing at all compression ratios. It is also clear that the higher the compression ratio used on our test set, the more robust the 3D U-Net is with respect do the 2D U-Net. Concerning the 2D U-Net segmentation of the bladder, for an acceptable mean DSC threshold of 0.7, we can compress up to a ratio of 64:1 and 48:1 using CT and CBCT scans respectively. Whereas for the 3D U-Net segmentation of the bladder, for an acceptable mean DSC threshold of 0.7, we can compress up to a ratio of 96:1 and 64:1 using CT and CBCT scans respectively. This means that we can compress our test sets up to 1.5 times as much for the same segmentation performances of the bladder. Similar observations can be made for the rectum segmentation. Concerning the 2D U-Net segmentation of the rectum, for an acceptable mean DSC threshold of 0.7, we can compress up to a ratio of 32:1 using both CT and CBCT scans. Whereas for the 3D U-Net segmentation of the rectum, for an acceptable mean DSC threshold of 0.7, we can compress up to a ratio of 48:1 using both CT and CBCT scans. This means that we can compress our test sets up to 1.5 times as much for the same segmentation performances of the rectum.

**Fig. 6:**
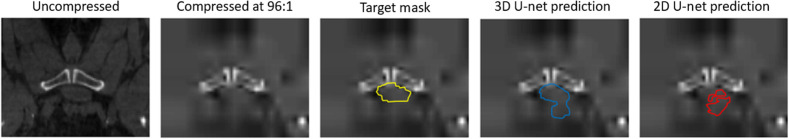
Experiment 1—Visual results for bladder segmentation using 2D and 3D U-Net with a compression ratio of 96:1 on a CBCT scan.

**Fig. 7:**
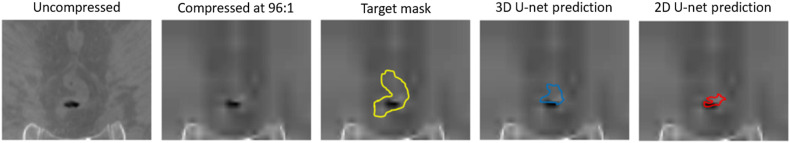
Experiment 1—Visual results for rectum segmentation using 2D and 3D U-Net with a compression ratio of 96:1 on a CBCT scan.

Referring to Figs. 6 and 7, we can observe the visual results with respect to the original targeted segmentation mask for a CBCT scan of the bladder and rectum at a compression ratio of 96:1. We can see that the 3D U-Net provides clearer outlines of the organs. We can also observe that the 2D U-Net is more likely to yield discontinuities in its segmentation which causes worst SMBD performances.

### 3.2 Experiment 2: 3D Versus 3D Fine-Tuned U-Net Robustness to JPEG 2000 Compression Trained at Several Compression Ratio and Tested at the Same Compression Ratios

In this experiment, the training phase is split into two phases. In the first phase, we train the 3D U-Net on uncompressed images similar to Experiment 1. In the second phase, we add an additional training phase by adding to the training set compressed/decompressed images. We do this for several compression ratios ranging from 24:1 to 128:1 and test the network on the same compression ratio as the one used for the second training phase. We then look at two segmentation evaluation metrics the DSC and SMBD in the case of both the CT and CBCT datasets on bladder and rectum segmentation. The detailed results of the experiment for both bladder and rectum segmentation are summarized in Tables 3 and 4 respectively.

**Table 3:** Experiment 2—Detailed DSC and SMBD performances after training and testing at several compression ratios for 3D and 3D fine-tuned U-Net segmentation of the bladder on CT and CBCT images.

Bladder		Compression ratio						
		1:1	24:1	32:1	48:1	64:1	96:1	128:1
DSC	3D FT CT	0.916±0.033	0.900±0.041	0.893±0.057	0.887±0.050	0.870±0.073	0.834±0.072	0.702±0.155
	3D CT	0.916±0.033	0.912±0.034	0.902±0.043	0.885±0.060	0.859±0.076	0.758±0.122	0.409±0.291
	3D FT CBCT	0.832±0.118	0.856±0.095	0.837±0.102	0.835±0.092	0.802±0.114	0.741±0.097	0.652±0.120
	3D CBCT	0.832±0.118	0.825±0.123	0.808±0.129	0.780±0.138	0.741±0.141	0.651±0.118	0.450±0.133
SMBD	3D FT CT	1.445±0.579	1.533±0.554	1.750±1.086	1.895±0.941	2.167±1.395	2.675±1.330	4.701±3.033
	3D CT	1.445±0.579	1.546±0.615	1.831±0.806	2.083±1.123	2.458±1.356	3.901±1.995	8.205±4.186
	3D FT CBCT	2.582±1.963	2.185±1.548	2.529±1.782	2.537±1.598	2.971±1.976	3.570±1.577	4.789±1.815
	3D CBCT	2.582±1.963	2.695±2.033	2.955±2.167	3.361±2.364	3.857±2.371	4.902±1.920	7.811±2.151

**Table 4:** Experiment 2—Detailed DSC and SMBD performances after training and testing at several compression ratios for 3D and 3D fine-tuned U-Net segmentation of the rectum on CT and CBCT images.

Rectum		Compression ratio						
		1:1	24:1	32:1	48:1	64:1	96:1	128:1
DSC	3D FT CT	0.771±0.068	0.762±0.082	0.763±0.073	0.739±0.082	0.729±0.066	0.667±0.069	0.516±0.158
	3D CT	0.771±0.068	0.772±0.066	0.773±0.061	0.752±0.062	0.709±0.063	0.617±0.094	0.373±0.186
	3D FT CBCT	0.766±0.073	0.778±0.079	0.763±0.085	0.756±0.080	0.729±0.095	0.649±0.106	0.572±0.130
	3D CBCT	0.766±0.073	0.765±0.070	0.748±0.078	0.711±0.097	0.647±0.120	0.471±0.114	0.272±0.135
SMBD	3D FT CT	2.653±1.029	3.236±2.110	3.272±2.058	3.467±2.074	3.239±1.552	4.038±1.407	4.602±1.903
	3D CT	2.653±1.034	2.642±1.056	2.602±0.967	2.862±1.146	3.197±1.065	4.322±2.432	8.917±4.143
	3D FT CBCT	2.348±0.867	2.388±1.094	2.644±1.170	2.807±1.184	2.614±0.902	3.261±0.903	3.920±0.867
	3D CBCT	2.348±0.867	2.308±0.802	2.413±0.823	2.604±0.952	3.245±1.593	4.935±1.689	9.404±2.837

**Fig. 8:**
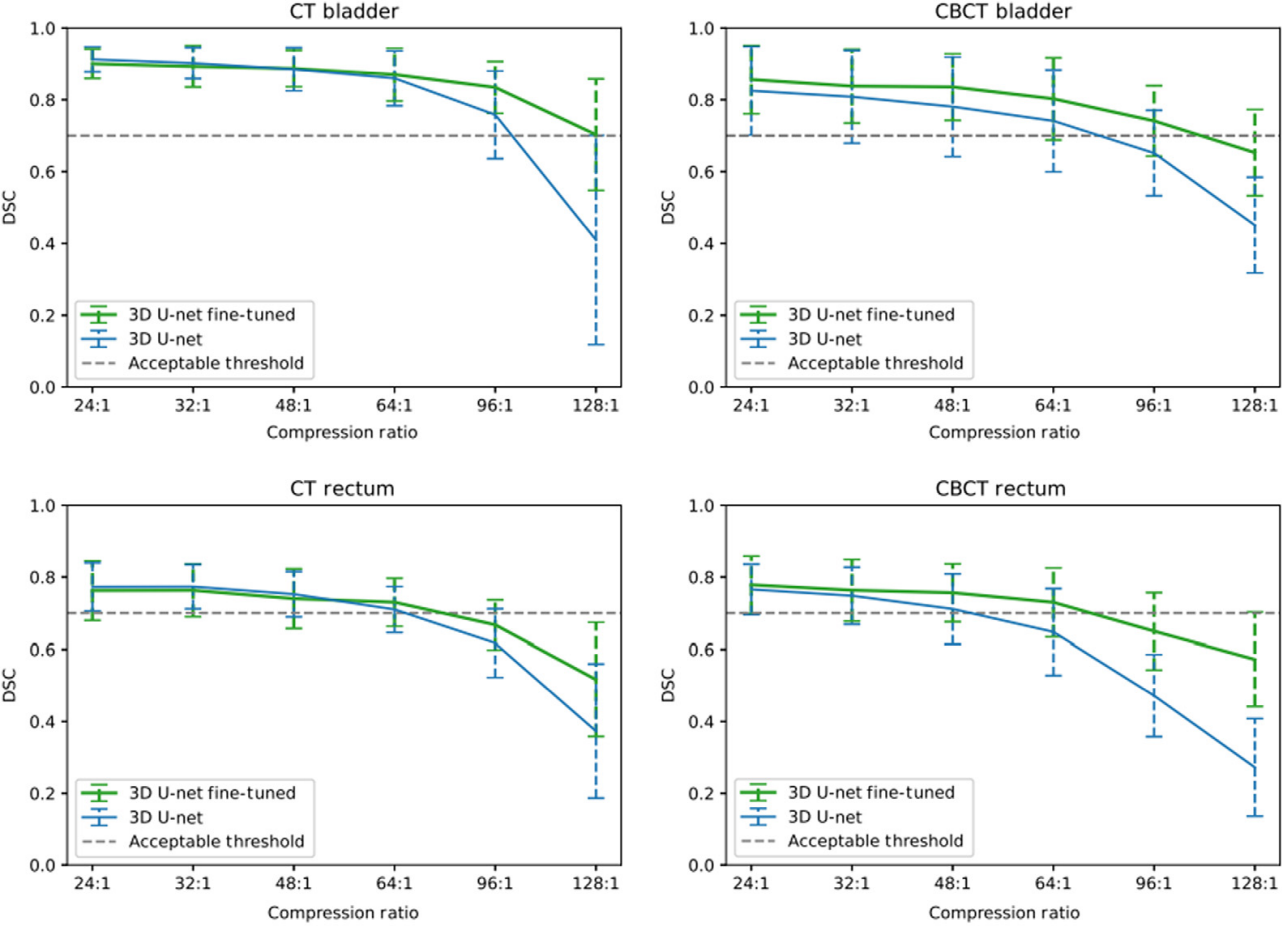
Experiment 2—DSC performances after training and testing at several compression ratios for 3D (blue) and 3D fine-tuned (green) U-Net segmentation of the bladder (top) and the rectum (bottom) on CT (left) and CBCT (right) images.

Referring to Fig. 8, we can observe better overall performance of the 3D fine-tuned U-Net network in terms of the DSC when testing at all compression ratios. It is also clear that the higher the compression ratio used on our test set, the more robust the 3D fine-tuned U-Net is with respect do the 3D U-Net. This is expected given our network has gone through a second training phase with compressed images. Concerning the 3D U-Net segmentation of the bladder, for an acceptable mean DSC threshold of 0.7, we can compress up to a ratio of 96:1 and 64:1 using CT and CBCT scans respectively. Whereas for the 3D fine-tuned U-Net segmentation of the bladder, for an acceptable mean DSC threshold of 0.7, we can compress up to a ratio of 128:1 and 96:1 using CT and CBCT scans, respectively. This means that we can compress our test sets up to 1.5 times as much for the same segmentation performances of the bladder. Similar observations can be made for the rectum segmentation. Concerning the 3D U-Net segmentation of the rectum, for an acceptable mean DSC threshold of 0.7, we can compress up to a ratio of 48:1 using both CT and CBCT scans. Whereas for the 3D fine-tuned U-Net segmentation of the rectum, for an acceptable mean DSC threshold of 0.7, we can compress up to a ratio of 64:1 using both CT and CBCT scans. This means that we can compress our test sets up to 1.5 times as much for the same segmentation performances of the rectum. When comparing the results of 3D fine-tuned U-Net from Experiment 2 to the 2D U-Net from Experiment 1, we can deduce that we can compress our test sets up to twice as much for the same segmentation performances of both the rectum and the bladder using CT and CBCT scans.

**Fig. 9:**
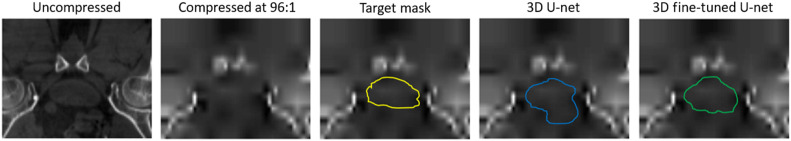
Experiment 2—Visual results for bladder segmentation using 3D and 3D fine-tuned U-Net with a compression ratio of 96:1 on a CBCT scan.

**Fig. 10:**
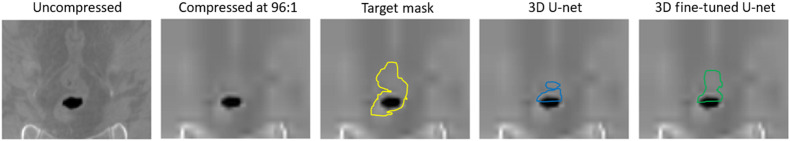
Experiment 2—Visual results for rectum segmentation using 3D and 3D fine-tuned U-Net with a compression ratio of 96:1 on a CBCT scan.

Referring to Fig. 9 and Fig. 10, we can observe the visual results with respect to the original targeted segmentation mask for a CBCT scan of the bladder and rectum at a compression ratio of 96:1. We can see that the 3D fine-tuned U-Net provides clearer outlines of the organs. We can also observe that the 3D U-Net is more likely to yield some discontinuities in its segmentation which causes worst SMBD performances.

### 3.3 Experiment 3: 3D Fine-Tuned U-Net Robustness to JPEG 2000 Compression Trained at Specific Compression Ratios and Tested at Several Compression Ratios

In this experiment, we find the ideal compression ratio at which we should train the 3D U-Net to ensure robustness to JPEG 2000 compression for testing at several compression ratios. Similar to Experiment 2, the training phase is split into two phases. The first phase is done with uncompressed images and the second training phase is done by adding compressed images at a specific compression ratio (48:1, 64:1, and 96:1) and testing the network at several compression ratios ranging from 24:1 to 128:1. We then look at two segmentation evaluation metrics the DSC and SMBD in the case of both the CT and CBCT datasets on bladder and rectum segmentation. The detailed results of the experiment for both bladder and rectum segmentation are summarized in Table 5 and Table 6, respectively.

**Table 5:** Experiment 3—Detailed DSC and SMBD performances after training at specific compression ratios of 48:1, 64:1, and 96:1 and testing at several compression ratios for 3D U-Net segmentation of the bladder on CT and CBCT images.

Bladder		Compression ratio						
		1:1	24:1	32:1	48:1	64:1	96:1	128:1
DSC	3D FT 48 CT	0.912±0.024	0.905±0.037	0.900±0.040	0.887±0.050	0.857±0.075	0.738±0.139	0.339±0.302
	3D FT 64 CT	0.901±0.015	0.904±0.035	0.899±0.040	0.889±0.051	0.870±0.073	0.779±0.144	0.406±0.310
	3D FT 96 CT	0.892±0.057	0.883±0.047	0.881±0.047	0.877±0.045	0.870±0.046	0.834±0.072	0.605±0.205
	3D FT 48 CBCT	0.874±0.071	0.862±0.084	0.853±0.087	0.835±0.092	0.797±0.101	0.685±0.112	0.414±0.155
	3D FT 64 CBCT	0.851±0.087	0.849±0.095	0.840±0.100	0.826±0.106	0.802±0.114	0.728±0.106	0.483±0.144
	3D FT 96 CBCT	0.823±0.102	0.814±0.099	0.807±0.102	0.796±0.106	0.780±0.111	0.741±0.097	0.592±0.104
SMBD	3D FT 48 CT	1.420±0.512	1.531±0.627	1.647±0.710	1.895±0.941	2.325±1.388	3.861±2.134	9.339±5.203
	3D FT 64 CT	1.501±0.554	1.594±0.594	1.694±0.731	1.892±0.970	2.167±1.395	3.372±2.203	8.712±4.890
	3D FT 96 CT	1.845±0.645	1.926±0.609	1.959±0.555	2.015±0.575	2.103±0.737	2.675±1.330	6.046±3.676
	3D FT 48 CBCT	2.012±1.287	2.110±1.348	2.248±1.454	2.537±1.598	2.978±1.686	4.028±1.337	7.555±2.211
	3D FT 64 CBCT	2.275±1.564	2.366±1.613	2.487±1.708	2.681±1.852	2.971±1.976	3.685±1.598	6.968±2.140
	3D FT 96 CBCT	2.685±1.495	2.795±1.510	2.946±1.652	3.093±1.813	3.276±1.936	3.570±1.577	5.338±1.641

**Table 6:** Experiment 3—Detailed DSC and SMBD performances after training at specific compression ratios of 48:1, 64:1, and 96:1 and testing at several compression ratios for 3D U-Net segmentation of the rectum on CT and CBCT images.

Rectum		Compression ratio						
		1:1	24:1	32:1	48:1	64:1	96:1	128:1
DSC	3D FT 48 CT	0.740±0.086	0.741±0.082	0.744±0.079	0.739±0.082	0.725±0.082	0.653±0.077	0.389±0.154
	3D FT 64 CT	0.740±0.070	0.740±0.067	0.742±0.062	0.737±0.063	0.729±0.066	0.675±0.067	0.416±0.155
	3D FT 96 CT	0.719±0.067	0.717±0.068	0.716±0.066	0.710±0.063	0.703±0.062	0.667±0.069	0.483±0.155
	3D FT 48 CBCT	0.758±0.099	0.766±0.087	0.764±0.079	0.756±0.080	0.731±0.086	0.607±0.112	0.385±0.128
	3D FT 64 CBCT	0.743±0.109	0.748±0.100	0.748±0.091	0.742±0.092	0.729±0.095	0.638±0.116	0.444±0.130
	3D FT 96 CBCT	0.700±0.107	0.700±0.104	0.699±0.099	0.698±0.096	0.682±0.099	0.649±0.106	0.538±0.128
SMBD	3D FT 48 CT	3.441±1.739	3.349±1.757	3.291±1.911	3.467±2.074	3.599±2.169	4.454±2.269	10.33±4.477
	3D FT 64 CT	3.231±1.080	3.167±1.099	3.111±1.121	3.212±1.205	3.239±1.552	4.013±1.854	9.504±4.106
	3D FT 96 CT	3.125±0.872	3.114±0.919	3.117±0.877	3.109±0.856	3.413±0.985	4.038±1.407	7.637±3.080
	3D FT 48 CBCT	2.734±1.272	2.608±1.222	2.582±1.176	2.807±1.184	2.708±1.040	4.789±2.993	10.67±3.940
	3D FT 64 CBCT	2.738±1.250	2.658±1.169	2.595±1.102	2.754±1.064	2.614±0.902	3.552±1.037	8.525±2.639
	3D FT 96 CBCT	3.179±1.095	3.168±1.078	3.120±1.050	3.050±0.954	3.121±0.927	3.261±0.903	5.880±1.535

**Fig. 11:**
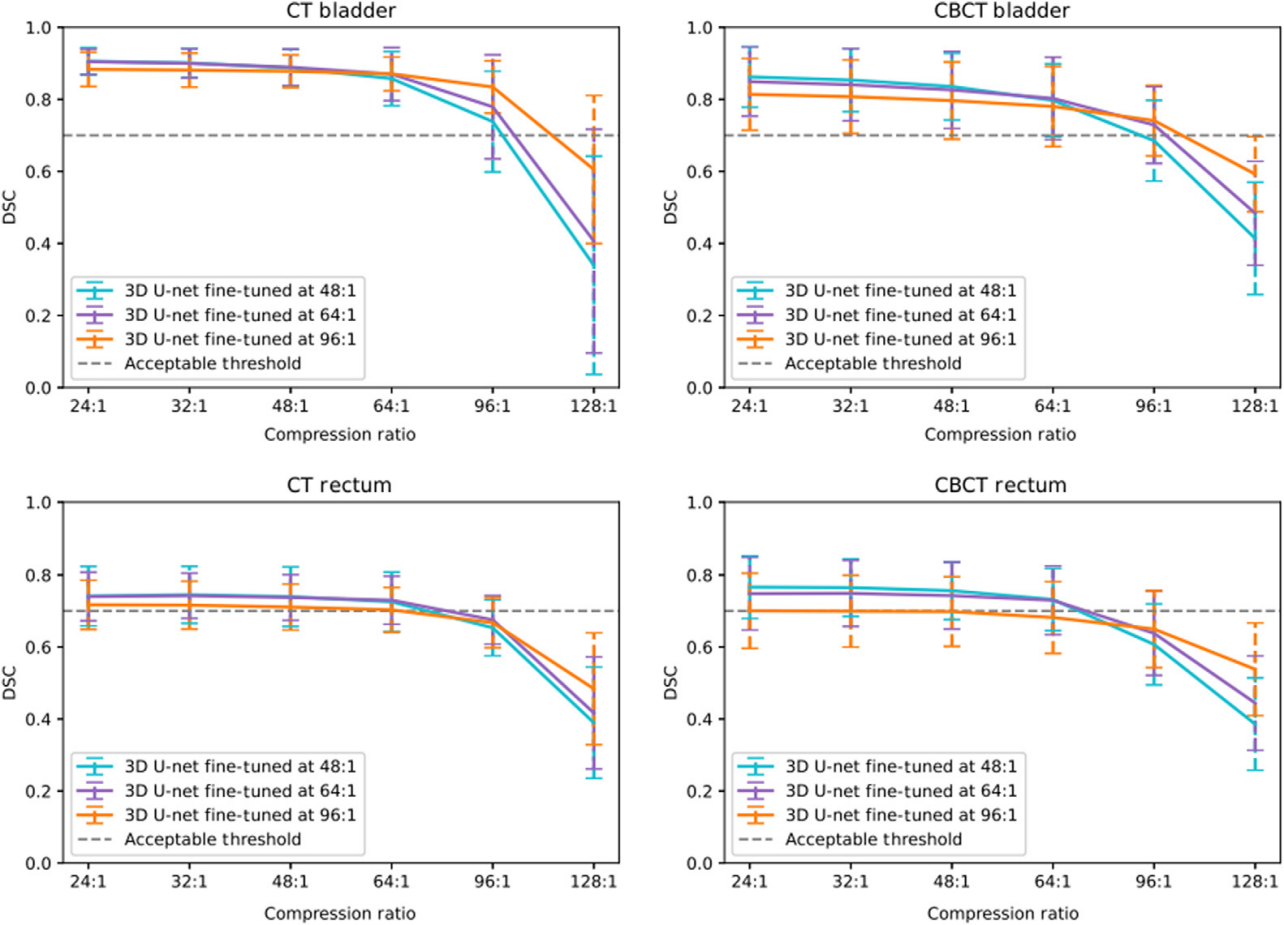
Experiment 3—DSC performances after training at specific compression ratios of 48:1 (cyan), 64:1 (purple) and 96:1 (orange) and testing at several compression ratios for 3D U-Net segmentation of the bladder(top) and the rectum(bottom) on CT (left) and CBCT (right) images

We observe the results when training our network in the second phase on only images compressed at 48:1, 64:1, and 96:1, respectively. We observe that the higher the compression on which our network is trained, the more robust it is to testing at high compression ratios. However, this also means that the network becomes weaker when testing at low compression ratios. Referring to Fig. 11, we can see that when training our network with a compression ratio of 48:1 and 64:1 we are able to maintain higher segmentation performance than at 96:1 however, the later provides higher segmentation performance at high compression ratios up to 128:1. Given the observations, to guarantee the flexibility of our network, we want to ensure that we retain high performance for all compression ratios up to the highest compression ratio possible. Given the results and setting a minimum mean DSC performance threshold of 0.7, we deduce that training our network at a compression ratio of 96:1 for bladder segmentation would allow this flexibility while guaranteeing high segmentation performance when compressing our test set at a compression ratio of 96:1 or lower. On the other hand, the same observation can be made at a compression ratio of 64:1 for rectum segmentation. Given that we are looking to guarantee high segmentation performance for the highest compression ratio possible for both the bladder and the rectum, a compression ratio of 64:1 would be the maximum compression ratio that would suit both. Therefore, for a network trained on a compression ratio of 64:1, we would ensure high segmentation performance for both organs for test sets compressed at a ratio of 64:1 or lower.

**Fig. 12:**
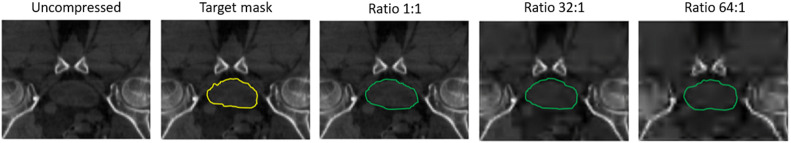
Experiment 3—Visual results for bladder segmentation using 3D fine-tuned U-Net trained with a compression ratio of 64:1 and tested at compression ratios of 1:1, 32:1, and 64:1 on a CBCT scan.

**Fig. 13:**
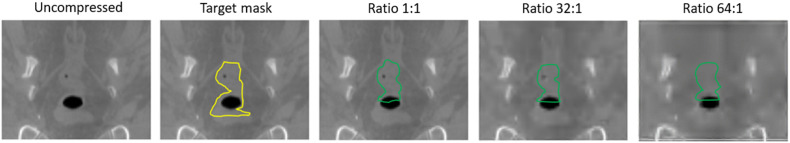
Experiment 3—Visual results for rectum segmentation using 3D fine-tuned U-Net trained with a compression ratio of 64:1 and tested at compression ratios of 1:1, 32:1, and 64:1 on a CBCT scan.

Referring to Figs. 12 and 13, we can observe the visual results with respect to the original targeted segmentation mask for a CBCT scan of the bladder and rectum when the 3D fine-tuned network is trained at a compression ratio of 64:1 and tested at compression ratios of 1:1, 32:1, and 64:1. We can observe successful segmentation of both the bladder and the rectum in all three cases underlining the robustness of proposed training strategy for our network.

## 4 Discussion

### 4.1 2D v 3D U-Net Robustness to Compression

**Fig. 14:**
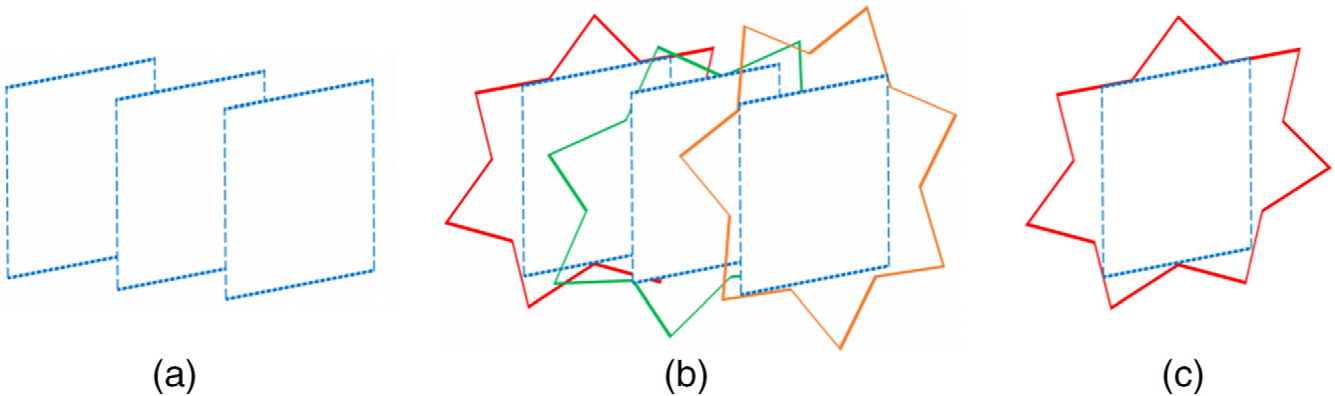
3D denoising representation. (a) Rectangular parallelepiped divided in slices; (b) independent noise added to the borders of each slice; (c) individual noisy slice.

In Experiment 1, we show that the 3D U-Net is more robust to compression than the 2D U-Net. This can be explained by analyzing the distortion created by the JPEG 2000 artifacts in the 2D and 3D compression case. In the case of 2D compression, given that we take the 3D volume and compress it slice by slice, the distortion caused by JPEG 2000 is independent from slice to slice. This means that two adjacent slices undergo a different distortion and therefore can have different results. In the case of the 3D compression, the 3D U-Net allows to average out the noise between the slices and recognize the original shape of the organ. This can visualized in Fig. 14. We can see that reconstructing the original parallelepiped shape from noisy rectangular slices is easier when using several noisy 2D slices rather than using individual 2D noisy slices.

### 4.2 Research Positioning

Positioning the research put forward in this paper is twofold. The first goal achieved by the paper is to validate the novel approach of studying the impact of compressed datasets on training and testing of 2D and 3D U-Net segmentation in a medical imaging context. The second goal is to encourage the use of compressed datasets in medical imaging by introducing a robust CNN that maintains high segmentation performance for 3D medical images at high compression ratios.

With regards to the chosen experimental methodology, in Experiment 2 and Experiment 3, we fine-tune our network by adding a second training phase using compressed images. As shown by Kim et al.^14^ and Svoboda et al.,^15^ we can train a CNN to recognize compression artifacts and correct them. Similarly, in Experiment 2 and Experiment 3, fine-tuning the CNN will teach it to adapt to compression effects and maintain successful segmentation performance by filtering the compression distortion in the deep features. Having a second training phase with purely compressed images, similar to the experiments done by Zanjani et al.^7^ for a classification task, proves to be highly successful at high compression ratios for the segmentation task. The network indeed specialize itself to extreme artifacts cause by compression and is able to maintain its high segmentation performance. This indeed shows that this methodology can be applied, in the context of medical imaging, not only to CNN trained for high-performance classification tasks but also to U-Net CNN for high-performance segmentation tasks. Even though our experimental setups differ from the work of Sharma et al.,^8^ we observe similar outcomes to theirs in our Experiment 2. In their work, they train their network on pure compressed data whereas we only add the compressed data at the fine-tuning phase. They also test their network on uncompressed data to see the utility of adding compressed data to the training set, whereas we test it on compressed data to observe the U-Net’s robustness to compression. However, we both come to the similar conclusion that a network trained with a compressed dataset outperforms networks trained on uncompressed datasets. We therefore both observe that compressing our training data has a data augmentation effect on our CNN.

Concerning the obtained compression robustness, in Experiment 1, when training our 3D U-Net on an uncompressed dataset an testing it on a test set at several compression ration, we observe a compression cutoff threshold up to 48:1 (included) for successful segmentation of both the bladder and the rectum. This cutoff threshold puts our compression tolerance twice above the works of Krupinski et al.^4^ and Zanjani et al.^7^ who can tolerate of compression threshold of 24:1 (included) in the same scenario as Experiment 1. When specializing our network by training it on a dataset with specific compression ratio of 64:1 we observe a compression cutoff threshold up to 48:1 (included) for successful segmentation of both the bladder and the rectum. This cutoff threshold puts us slightly above the compression threshold achieved by Zanjani et al.^7^ of 48:1 (included) in the same scenario as Experiment 3.

### 4.3 Limitations

The main limitation that we faced is defining the adequate evaluation criteria for the assessment of our results. We understand that the DSC is a mathematical evaluation criterion, therefore it is possible in some cases for two images to have the same DSC for the same organ but have two different clinical evaluations by the medical practitioner. In this paper, we defined an empirical minimum DSC threshold of 0.7. This threshold has been validated by our research team and the original team of annotation experts after observing several visual outcomes of U-Net organ segmentation results on our dataset. The 0.7 DSC threshold has also been validated by Carillo et al.^16^ following their work on organ segmentation in the male pelvic region. They found 0.7 as an acceptable DSC threshold following a quantitative assessment study involving 15 physicians that performed penile bulb contouring on CT scans of 10 patients suffering from possible erectile dysfunction and urinary toxicity after radiotherapy for prostate cancer. We understand that the DSC threshold may differ from one organ to another for correct segmentation. But given that both our works involve contouring organs on CT scans in the male pelvic region, we feel comfortable using it as additional validation for our DSC threshold. Nevertheless, the choice of the minimum DSC threshold in our work is key as it is inversely proportional to the compression cut off threshold. Another potential limitation is that the DSC does not show whether or not the segmentation has impacted the medical practitioner decision making in either the treatment planning or the treatment delivery phases. In our case, the images are not only used for contouring purposes, but are also used to calculate the exact dose to be delivered to the patient. Ideally, you would like to set the compression cut off threshold based on whether or not the slight deterioration in the image due to compression impacted the medical practitioner’s decision making. However, this evaluation method is subjective, hard to quantify, and time consuming.

## 5 Conclusion and Future Work

With the emergence of deep learning applications in medical imaging, the challenge of having large amounts of readily available data has made image compression a necessity. This has led researchers in the field to look into the impact of image compression on the performance of deep learning algorithms in medical imaging as well as its impact on the diagnostic capabilities of medical practitioners. In this paper, we extended the work done in the field on the impact of image compression on the performance of deep learning-based image classification, to evaluating the effect of image compression on the performance of deep learning-based image segmentation. More precisely, we have studied the impact of JPEG 2000 compression on 2D and 3D CNN-based U-Net segmentation of male pelvic organs. We conducted three experiments on 56 CBCT and 74 CT scans to improve U-Net performance against JPEG 2000 compression for bladder and rectum segmentation. We have shown that 3D U-Net segmentation is more robust to compression that a 2D U-Net. Furthermore, by fine-tuning the 3D U-Net, we can go up to a compression ratio twice as high for the same segmentation performance compared to a 2D U-Net. Moreover, we determine that fine-tuning our network to a specific compression ratio of 64:1 will ensure its flexibility to be used at compression ratios equal or lower with high segmentation performance. This work is to be seen as a positive result to further encourage the use of image compression in the medical imaging field.

To increase the validation of our research, future work should be extended to tackle various applications in medical imaging, which rely on 3D U-Net segmentation of organs based on CTs and/or CBCTs. This can range from segmentation of lung nodules for detection of lung carcinoma^17^ to kidney segmentation for treatment of renal cell carcinoma^18^ and whole-heart segmentation to aid early detection of critical cardiovascular diseases.^19^ Another interesting path that we are currently looking into is to incorporate compressed images directly into the 3D U-Net segmentation. Gueguen et al.^20^ investigated the potential of using the blockwise DCT coefficients of the JPEG codec and training the CNN directly on them. This method has the potential to be faster and more accurate than most traditional CNNs, by reducing the network’s complexity and therefore decreasing random access memory (RAM) consumption. In our case, we would need to adapt the 3D U-Net to work directly on the DWT coefficients of the JPEG2000 codec which should induce even better results given the flexibility of JPEG2000.

## References

[1] Schreier J. et al., “Clinical evaluation of a full-image deep segmentation algorithm for the male pelvis on cone-beam CT and CT,” Radiother. Oncol. 145, 1–6 (2020). RAONDT 0167-8140 10.1016/j.radonc.2019.11.021 31869676

[2] Léger J. et al., “Cross-domain data augmentation for deep-learning-based male pelvic organ segmentation in cone beam CT,” Appl. Sci. 10(3), 1154 (2020). 10.3390/app10031154

[3] Kalinski T. et al., “Lossless compression of JPEG2000 whole slide images is not required for diagnostic virtual microscopy,” Am. J. Clin. Pathol.y 136(6), 889–895 (2011). AJCPAI 0002-9173 10.1309/AJCPYI1Z3TGGAIEP 22095374

[4] Krupinski E. et al., “Compressing pathology whole-slide images using a human and model observer evaluation,” J. Pathol. Inf. 3(1), 17 (2012). 10.4103/2153-3539.95129 PMC3352607 22616029

[5] Pantanowitz L. et al., “Impact of altering various image parameters on human epidermal growth factor receptor 2 image analysis data quality,” J. Pathol. Inf. 8(1), 39 (2017). 10.4103/jpi.jpi_46_17 PMC5609390 28966838

[6] Marcelo A. et al., “Effect of image compression on telepathology: a randomized clinical trial,” Arch. Pathol. Lab. Med. 124(11), 1653–1656 (2000). 10.1043/0003-9985(2000)124<1653:EOICOT>2.0.CO;2 11079019

[7] Zanjani F. G. et al., “Impact of JPEG 2000 compression on deep convolutional neural networks for metastatic cancer detection in histopathological images,” J. Med. Imaging 6(2), 027501 (2019). JMEIET 0920-5497 10.1117/1.JMI.6.2.027501 PMC6479230 31037247

[8] Sharma S. et al., “Performance analysis of semantic segmentation algorithms trained with JPEG compressed datasets,” Proc. SPIE 11401, 1140104 (2020). PSISDG 0277-786X 10.1117/12.2557928

[9] Auli-Llinas F. Serra-Sagrista J., “JPEG2000 quality scalability without quality layers,” IEEE Trans. Circuits Syst. Video Technol. 18(7), 923–936 (2008). 10.1109/TCSVT.2008.920748

[10] Herrmann M. et al., “Implementing the DICOM standard for digital pathology,” J. Pathol. Inf. 9(1), 37 (2018). 10.4103/jpi.jpi_42_18 PMC6236926 30533276

[11] Zhi X., “UNet for images,” 2017, https://github.com/zhixuhao/unet.

[12] Brion E., “Pelvis segmentation,” 2020, https://github.com/eliottbrion/pelvis_segmentation.

[13] Ronneberger O. Fischer P. Brox T., “U-Net: convolutional networks for biomedical image segmentation,” Lect. Notes Comput. Sci. 9351, 234–241 (2015). LNCSD9 0302-9743 10.1007/978-3-319-24574-4_28

[14] Kim T. et al., “SF-CNN: a fast compression artifacts removal via spatial-to-frequency convolutional neural networks,” in IEEE Int. Conf. Image Process. (2019). 10.1109/icip.2019.8803503

[15] Svoboda P. et al., “Compression artifacts removal using convolutional neural networks,” J. WSCG 24, 63–72 (2016).

[16] Carillo V. et al., “Contouring variability of the penile bulb on CT images: quantitative assessment using a generalized concordance index,” Int. J. Radiat. Oncol. Biol. Phys. 84(3), 841–846 (2012). IOBPD3 0360-3016 10.1016/j.ijrobp.2011.12.057 22401919

[17] Xiao Z. et al., “Segmentation of lung nodules using improved 3D-UNet neural network,” Symmetry 12(11), 1787 (2020). SYMMAM 2073-8994 10.3390/sym12111787

[18] Zhao W. et al., “Mss U-Net: 3D segmentation of kidneys and tumors from CT images with a multi-scale supervised u-net,” Inf. Med. Unlocked 19, 100357 (2020). 10.1016/j.imu.2020.100357

[19] Habijan M. et al., “Whole heart segmentation from CT images using 3D U-Net architecture,” in Int. Conf. Syst. Signals Image Process. (2019). 10.1109/iwssip.2019.8787253

[20] Gueguen L. et al., “Faster neural networks straight from JPEG,” in Advances in Neural Information Processing Systems, Bengio S. et al., Eds., Vol. 31, Curran Associates, Inc. (2018).

